# A new species of *Herpetogramma* (Lepidoptera, Crambidae, Spilomelinae) from eastern North America

**DOI:** 10.3897/zookeys.149.2344

**Published:** 2011-11-24

**Authors:** Louis Handfield, Daniel Handfield

**Affiliations:** 1 845, de Fontainebleau, Mont-Saint-Hilaire, Québec, Canada, J3H 4J2; 2 355, Chemin des Grands Coteaux, Saint-Mathieu-de-Beloeil, Québec, Canada, J3G 2C9

**Keywords:** Taxonomy, *Herpetogramma*, Crambidae, eastern North America, Dryopteridaceae, Christmas fern, *Polystichum*

## Abstract

*Herpetogramma sphingealis* **sp. n.**, a new species of Crambidae (Lepidoptera), is described from Québec, Canada. The species is included in the genus *Herpetogramma* Led., 1863, a genus in the subfamily Spilomelinae. Adults and genitalia of this species are described and illustrated, as well as those of *Herpetogramma aeglealis* (Walker, 1859) and *Herpetogramma thestealis* (Walker, 1859), and adults of the semi-melanic form of *Herpetogramma aeglealis* are illustrated.

## Introduction

On the 21st day of July 2004, the authors were collecting moths at light on the slopes of Mont Rougemont at Rougemont, in the Province of Québec. We were surprised by the significant numbers of a dark moth, which we identified readily as a pyraloid, but a pyraloid that was new to us. Though pyraloids were not our first goal, we decided to collect a few, considering their number and oddity.

One of the authors (LH) showed the moths to Eugene Munroe who identified them as a form of *Herpetogramma aeglealis* (Walker, 1859). We were still in doubt because the moths were much larger than normal *Herpetogramma aeglealis* and because this form had never been found in Québec before, as confirmed by searching in our personal collections (DH, LH), and those of Ouellet-Robert (Université de Montréal), the Lyman Museum (McGill University), and the CNC at Ottawa. Collectively, these collections had more than 195 specimens of *Herpetogramma aeglealis* collected between 1906 and 2000 in Québec, but the larger, dark form was not found in any of these collections.

Following that finding, we discovered on the Internet (Maryland moths, www.marylandmoths.com [accessed September 2011]) photographs of two moths looking very similar to our specimens. According to John Glaser, the specimens were a new species of *Herpetogramma* Lederer, in which the larvae feed on Christmas fern (Dryopteridaceae: *Polystichum acrostichoides* (Michx.) Schott). Dr Glaser wrote to the first author (LH, in litt., 11.viii.2008) that he based his conclusion on a note written to him by the late Douglas C. Ferguson.

We then decided to look further in that matter. The field season of 2008 permitted us to collect more than 75 specimens of both sexes. At the same time, we also collected 77 specimens of *Herpetogramma aeglealis* from the same localities. We also found that Christmas fern was common in all the sites (Rougemont, Otterburn Park, Mont-Saint-Hilaire, etc.) where large dark specimens were found. Moreover, the moth was not seen in other sites where Christmas fern was absent, but where typical *Herpetogramma aeglealis* occurred.

We were pleased that Don Lafontaine of the CNC showed some interest and decided to make genitalic dissections of both forms, including a rarely-seen semi-melanic form of *Herpetogramma aeglealis*, and specimens of the closely related species *Herpetogramma thestealis* (Walker, 1859). The genitalia did not show significant differences, except for the aedeagus and vesica, which did show constant differences among the three species from various localities in Québec and eastern United States. This permitted us to confirm that the large, dark form was really a separate species from both *Herpetogramma aeglealis* and *Herpetogramma thestealis*, but it remained to be determined if there was an available name for this taxon. We also confirmed that the semi-melanic form was really a form of *Herpetogramma aeglealis* and not a form of the dark species or a separate species.

On the basis of the male genitalia, the new species is more closely related to *Herpetogramma aeglealis* than to *Herpetogramma thestealis*. In both the new species and *Herpetogramma aeglealis* the large spine-covered diverticulum is near the middle of the left side of the vesica, there is a double-pouched subbasal diverticulum on the right-ventral surface, and no subbasal diverticulum on the left. In *Herpetogramma thestealis* the large spine-covered diverticulum is about one-third from the apex of the vesica on the dorsal surface, there is only a single subbasal diverticulum on the left-ventral surface, and there is an additional, spined, subbasal diverticulum on the right side. Other characters of the genitalia are similar in the three species.

## Matherials and methods

### Repository abbreviations

Specimens were examined from the following collections:

AMNH American Museum of Natural History, New York, NY, USA

BMNH The Natural History Museum (statutorily, British Museum (Natural History)), London, UK

CNC Canadian National Collection of Insects, Arachnids, and Nematodes, Ottawa, Ontario, Canada.

CUIC Cornell University Insect Collection, Ithaca, New York, USA

DH Personal collection of Daniel Handfield, Saint-Mathieu-de-Beloeil, Québec, Canada

LH Personal collection of Louis Handfield, Mont-Saint-Hilaire, Québec, Canada

LMIC The Lyman Museum Insect Collection, McGill University, Ste-Anne-de-Bellevue,Québec, Canada

UMIC Ouellet-Robert collection, Université de Montréal, Montréal, Québec, Canada

USNM National Museum of Natural History (formerly, United States National Museum), Washington, District of Columbia, USA

### Dissecting methods and genital terminology

Dissection of genitalia and terms for genital structures and wing markings follow Lafontaine (2004).

### History, names and synonyms

The first author to deal with the many names included under the genus *Herpetogramma* was Forbes (Forbes 1923). He included the genus within a broader concept of the genus *Pyrausta* Schranck. It is clear from his text (p. 567) that his concept of *aeglealis* is exactly what we now know under that name. He has also referred to the semi-melanic form of *aeglealis*, but does not refer to a larger black form. Moreover, an examination by Don Lafontaine (pers. comm. 2010) of the Lepidoptera collection of the Cornell University (New York) (where Forbes was a professor) did not reveal any specimen referable to *Herpetogramma sphingealis*. After Forbes, more than a half century elapsed before Munroe (1983) transferred the species to *Herpetogramma* and reduced the many names to nine recognized species. Munroe listed only *Herpetogramma quinquelinealis* (Grote, 1875) in the synonym of *Herpetogramma aeglealis*. The original descriptions of both names (Walker 1859 and Grote 1875) have been consulted and it is evident that none refers to the black species herein described. Also, photographs of the type specimens of both species in the Natural History Museum, London (BMNH) were examined and it is evident that these types only refer to *Herpetogramma aeglealis* and not to the new species. Additionally, none of the European *Herpetogramma* species looks like the new species (Goater 1986; Slamka 2010), and no similar species exists in Japan (Esaki et al. 1970) or China (Pingyuan et al. 1981). The widespread occurrence of *Herpetogramma sphingealis* in eastern North America, which essentially follows that of its native host plant, Christmas fern, makes it unlikely that the species is introduced from abroad.

In the USNM, there is one specimen reared by Wilton Everett Britton on Christmas fern in New Haven, Connecticut, that emerged 16 July 1900; the larva was collected near Maltby Lakes, New Haven County, Connecticut. Charles Henry Fernald recognized it as a new species, put a manuscript name with the specimen, but the name was never published.

In a recent paper by Alma Solis (Solis 2010), the types and identities of the species of *Herpetogramma* in Canada and the United States are discussed, but there are no remarks that might pertain to the undescribed species are given under *Herpetogramma aeglealis*. However, a male of the new species is illustrated (Solis 2010, fig. 2) from Six Mile Creek near Ithaca New York, but without comment.

The new species will key out to *Herpetogramma aeglealis* in Solis (2010), but can be distinguished from it by the characters given in the following diagnosis and description.

## Taxonomy

### Herpetogramma sphingealis

Handfield and Handfield sp. n.

urn:lsid:zoobank.org:act:C32535D3-10D1-4F5D-B4EE-9E6E5405B732

http://species-id.net/wiki/Herpetogramma_sphingealis

Figs 1, 2, 8 11

#### Type material.

**Holotype** ♂. Rougemont Mountain, Rougemont, Québec (45°28'026"N, 73°04'029"W), 29.vii.2008, Daniel Handfield, MDH006041, CNC type No. 23981. CNC. **Paratypes** 83 ♂, 24 ♀: Mont-Saint-Hilaire, Québec, 20.vii.2003 (1 ♂), 20.vii.2008 (1 ♂), Louis Handfield; Otterburn Park, Bosquets Hudon, Québec, 31.vii.2009 (2 ♂), 5.viii.2008 (2 ♂, 2 ♀), Louis Handfield; Rougemont, mountain, Québec, 11.vii.2008 (1 ♂), 16.vii.2008 ( 2 ♂), 19.vii.2009 (1 ♂), 21.vii.2004 (1 ♂), 21.vii.2008 (5 ♂), 23.vii.2009 (3 ♂), 24.vii.2008 (2 ♂, 2 ♀), 25.vii.2008 (13 ♂, 3 ♀), 27.vii.2008 (5 ♂, 5 ♀), 28.vii.2009 (3 ♂, 1 ♀), 29.vii.2008 (10 ♂, 5 ♀), 30.vii.2009 (3 ♂), 1.viii.2008 (3 ♂), 3.viii.2009 (5 ♂, 2 ♀), 5.viii.2009 (4 ♂, 1 ♀), 7.viii.2009 (1 ♂), Louis Handfield; 21.vii.2004 (4 ♂), Daniel Handfield, 29.vii.2008 (8 ♂, 2 ♀), Daniel Handfield, 21.vii.2008 (1 ♂), Norman Handfield; Roxton Falls, Québec, 24.vii.2008 (2 ♂), Norman Handfield; St-Armand, Québec, 20.vii.2004 (1 ♀), 21.vii.2004 (1 ♂), Claude Chantal, in coll. Léo-Paul Landry; Ste-Anne-de-Bellevue, Morgan Arboretum, Québec, 12.viii.2009 (2 ♂), Louis Handfield, 12.viii.2009 (1 ♂), Daniel Handfield; Varennes, Québec, 7.vii.2008 (1 ♂), Claude Chantal, in coll. Michel Pratte).

#### Other material examined.

**USA.** Specimens were examined from the following states: Arkansas (USNM), Connecticut (AMNH, USNM), Delaware (USNM), Georgia (CNC), Kentucky (USNM), Louisiana (USNM), Maryland (USNM), Mississippi (USNM), New Jersey (USNM), New York (USNM), North Carolina (USNM), Pennsylvania (USNM), Tennessee (USNM) and Virginia (USNM).

#### Etymology.

The Latin name *sphingealis* refers to the sphingid-like appearance of the males.

#### Diagnosis.

*Herpetogramma sphingealis*, like *Herpetogramma aeglealis* and *Herpetogramma thestealis*, is sexually dimorphic. The male of *Herpetogramma sphingealis* is likely to be confused only with *Herpetogramma aeglealis*, but can be distinguished from *Herpetogramma aeglealis* by its nearly uniform dark-brown colour and large wingspan (34–37 mm versus 29–34 mm in *Herpetogramma aeglealis*). The transverse lines are obscure whereas in *Herpetogramma aeglealis* they are more sharply defined, usually with pale shading adjacent to them and with pale streaks between veins, especially in medial area. The hind wing is dark brown with a dark discal spot, but in *Herpetogramma aeglealis* the hindwing is dirty white with dark-gray shading on discal spot, wing veins, subterminal area, and an irregular but contrasting postmedial line. The female is larger than that of *Herpetogramma aeglealis* (31–34 mm versus 27–31 mm), has more apically-squared wings and is less uniformly dark coloured, so it resembles some females of *Herpetogramma aeglealis*, but females *Herpetogramma aeglealis* are paler, smaller, and always show a golden hue, never dark brown as in *Herpetogramma sphingealis*. Rare specimens of a semi-melanic form of *Herpetogramma aeglealis* have an overall dark coloring, as in *Herpetogramma sphingealis*, but the transverse lines are very well marked and followed by a larger creamy band, and also have a more extensive cream-colored shading in the costal area of the hindwing. In the male genitalia, *Herpetogramma sphingealis* differs from *Herpetogramma aeglealis* in having a longer aedeagus (10.0–10.6 × as long as the medial width compared to 8.0–8.8 × as long in *Herpetogramma aeglealis*) and also in details of vesica. In the female genitalia, *Herpetogramma sphingealis* has a longer ductus bursa (0.27 × as long as corpus bursae in *Herpetogramma sphingealis*, but only 0.22 x as long in *Herpetogramma aeglealis*).

#### Description.

Adult male:wingspan 34–37 mm (*Herpetogramma aeglealis* 29–34 mm). Upperside of head, palpi (excFept tufts at base), protothoracic collar, and upperside of thorax concolourous, chocolate brown, fading to a slightly paler brown with age; antennae filiform, finely ciliate on underside, each segment concolourous dorsally with upper surface of head; upperside of abdomen concolourous with wings, except for posterior brownish-yellow tuft covering valvae; maxillary palpi, legs, and underside of head, thorax, abdomen pure white; dark-brown band (nearly width of eye on side of head) and including the top of the maxillary palpi and chaetosema gives head appearance of having a longitudinal mask; eye black with greenish bands. Forewing chocolate brown, concolourous with upperside of head, thorax, abdomen, fading slightly to a paler brown; apex acutely angled; postmedian line slightly zigzagging from costa to halfway down wing, then turning abruptly inward at nearly right angle to position below reniform spot before turning downwards and zigzagging to posterior margin of wing; no other lines visible (except sometimes a vague trace of an outward-curved antemedian line); only other marks on forewing are a white patch on fringe at anal angle, two black dots at position of orbicular and the reniform spots, a cream-coloured rectangular patch between two black dots, and a dark terminal line at base of fringe; fringe concolourous with wing except for white anal patch and slightly darker shading on veins; fringes also fading with age. Hindwing concolourous with forewing, including fringe, fringe with dirty-white shading at anal angle; no lines visible; a round (more like a lunar crescent in *Herpetogramma aeglealis*) black discal dot with a creamy-white irregular patch toward wing base (nearly hidden by posterior margin of forewing). Fringes of all wings even, not crenate. Underside of all wings, including fringes, a dark grey, fading to a paler whitish grey toward wing base with white at base near pure-white thorax, especially along inner margin of hindwing; creamy patch and two black dots on forewing barely visible as is discal spot of hindwing. Legs mainly pure white, sometimes with brownish scales on upperside of anterior and posterior legs.

*Adult female*:wingspan 31–34 mm (*Herpetogramma aeglealis* 27–31 mm). Essentially same as for male except forewing larger, less elongated, and more square at margin; colour of wings a paler chocolate brown, transverse lines more contrasting. Hindwings as for male, but colour fading near base, sometimes showing a vague trace of a postmedian line.

*Genitalia*. Male genitalia of *Herpetogramma sphingealis* differ from those of *Herpetogramma aeglealis* in length of aedeagus and the details of vesica. In *Herpetogramma sphingealis* aedeagus long, 10.0–10.6 × as long as medial width compared to 8.0–8.8 × in *Herpetogramma aeglealis*. Also, secondary pouch on subbasal diverticulum broad and rounded, but narrow and finger-like in *Herpetogramma aeglealis*. Spinules on surface of the basal part of the vesica minute and difficult to see in *Herpetogramma sphingealis* but larger and more conspicuous in *Herpetogramma aeglealis*. Female genitalia similar to those of *Herpetogramma aeglealis*, except for length of ductus bursae (0.27 × as long as corpus bursae in *Herpetogramma sphingealis*, but only 0.22 × in *Herpetogramma aeglealis*), this likely reflecting longer aedeagus of *Herpetogramma sphingealis*. Genitalic dissections of specimens of dark semi-melanic forms allow specimens to be identified as *Herpetogramma aeglealis*.

**Figures 1–8.**
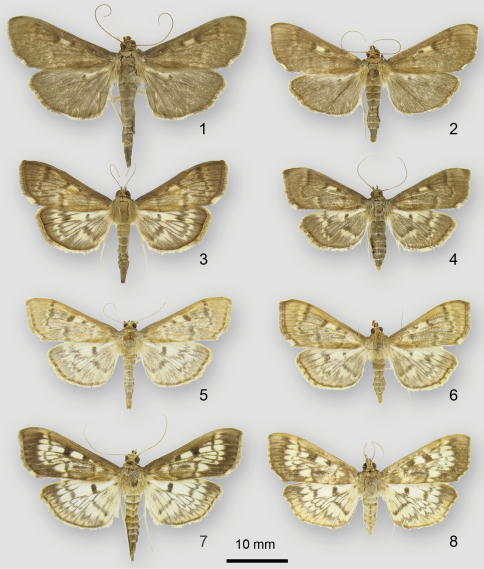
Adults of *Herpetogramma* species. **1** *Herpetogramma sphingealis*, male (Rougemont, Québec) **2** *Herpetogramma sphingealis*, female (Rougemont, Québec) **3** *Herpetogramma aeglealis*, male, dark form (Mont-St-Hilaire, Québec) **4** *Herpetogramma aeglealis*, female, dark form (Rougemont, Québec) **5** *Herpetogramma aeglealis*, male, typical form (Rougemont, Québec) **6** *Herpetogramma aeglealis*, female, typical form (Rougemont, Québec) **7** *Herpetogramma thestealis*, male (Rougemont, Québec) **8** *Herpetogramma thestealis*, female (Otterburn, Québec)

**Figures 9–14.**
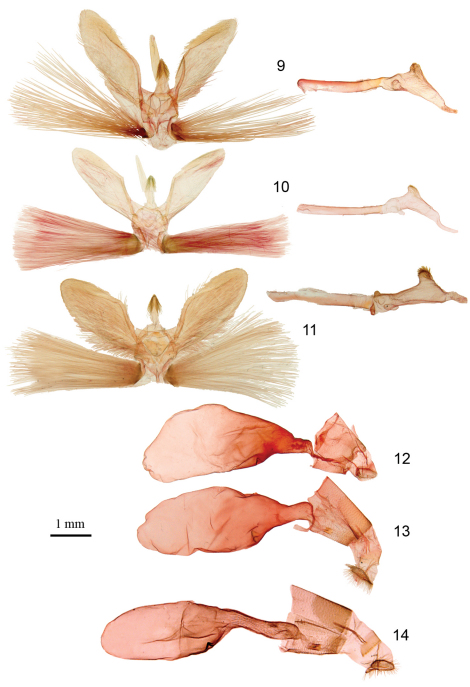
Genitalia of *Herpetogramma* species. **9** *Herpetogramma aeglealis*, male genitalia (Rougemont, Québec) **10** *Herpetogramma sphingealis*, male genitalia (Rougemont, Québec) **11** *Herpetogramma thestealis*, male genitalia (Rougemont, Québec) **12** *Herpetogramma aeglealis*, female genitalia (Rougemont, Québec) **13** *Herpetogramma sphingealis*, female genitalia (Rougemont, Québec) **14** *Herpetogramma thestealis*, female genitalia (Normandale, Ontario)

#### Biology and habitat.

*Herpetogramma sphingealis* occurs in the darkest areas of rich xeric forests, with maples and oaks, especially rocky, hilly, maple groves where Christmas fern occurs commonly. Its dark-brown colour is well suited for hiding in these woods. The moth comes readily to light and flies at the beginning of the night; it is sometimes one of the first to come to light. Its flight is darting and rapid. The underside of the head, thorax and abdomen, including the legs, are pure white, so it is easily spotted when flying to the light. The moth is a beautiful cryptically-coloured creature well-adapted to hide in the darkest shadows of the woods.

According to the “Moths of Maryland" (www.marylandmoths.com) and to specimens collected by Doug Ferguson in USNM, the larvae feed on Christmas fern. Doug Ferguson collected two larvae on “Xmas Fern" at “Richard Russell Pkwy, Union County, Georgia, 25 April 1969" and reared them with success on this host plant (note by Doug Ferguson to John Glaser, in litt., sent to the author [LH] 11 August.2008).

An additional specimen was reared as a leaf roller on Christmas fern and deposited in USNM was reared by Wilton Everett Britton and emerged on the 16 July 1900. Philip Dowell (1911) noticed in 1908 in New York State that the fronds of Christmas ferns and some of woodferns (*Dryopteris* Adans.) were attacked by a leaf roller, most probably by this new species for Christmas fern.

Larvae of a species of *Herpetogramma* were reared on Christmas fern in Athens, Georgia (Ruehlman et al. 1988); the species was identified as *Herpetogramma aeglealis*, but through the courtesy of Dr Matthews, one male specimen was loaned to the CNC, and a complete dissection including the vesica was prepared by Don Lafontaine, and it proved to be a specimen of the new species. All specimens for that study were determined to be a single species, due to their larvae, habits, host plant, and appearance of the adults, we believe they all belong to *Herpetogramma sphingealis* and not to *Herpetogramma aeglealis*. According to these authors, the larvae are solitary leaf rollers and live on the terminal leaflets of young fronds, passing the winter as eggs, emerging at the beginning of the spring and eating the fronds within a silk shelter for around a month, and then pupate to emerge about 12 days later.

David Wagner and his associates have reared two males and one female on Christmas fern in Connecticut. The males from Bridgewater, Litchfield Co., emerged on 12 June 1999 and 4 July1999 and a female from Chaplin, Windham Co., emerged on 30 July 2009. The specimens were identified by the author (LH).

As a result of these data, Christmas fern is the host plant of *Herpetogramma sphingealis*. It is possible that the larvae might be found on other species of ferns, although only one other species of the hollyfern genus (*Polystichum* Roth) has a range that overlaps that of *Herpetogramma sphingealis*. The range of the boreal species *Polystichum braunii* (Spenner) Fée, Braun's holly fern, overlaps that of *Herpetogramma sphingealis* in Québec and New England.

In contrast to the restricted host plant records for *Herpetogramma sphingealis*, both *Herpetogramma aeglealis* and *Herpetogramma thestealis* appear to be more general feeders as larvae. *Herpetogramma aeglealis* has been reared on a variety of herbaceous plants including ragwort [Asteraceae] and ferns [Polypodiales] (D. Wagner, pers. comm.), goldenrod [Asteraceae] and raspberry [Rosaceae] (Solis 2010), pokeweed (Phytolaccaceae) [Forbes 1923], and mayapple [Berberidaceae] (Judd 1954). *Herpetogramma thestealis* appears to be associated with woody plants, such as basswood [Tiliaceae] and hazel [Betulaceae] (Forbes 1923), and Carolina silverbell [Styraceae] and spikenard [Araliaceae] (D. Wagner, pers. comm. 2011).

#### Distribution.

*Herpetogramma sphingealis* occurs from southern Québec southward in eastern United States to Georgia and Louisiana and as far west as Arkansas. Christmas fern occurs from southeastern Canada southward to northern Florida and west to eastern Iowa and eastern Texas. At present, *Herpetogramma sphingealis* is known to occur over most of its host plant's range, and may occur over all of it.

The species seems to be expanding its distribution, at least to the North. It is spreading to new localities in southern Québec, appearing in some places that are well collected by the authors and colleagues where it had never been seen previously. For example, Mont-Saint-Hilaire has been collected by the author (LH) since 1966, Otterburn Park (Les Bosquets Hudon) since 1970 (LH), Ste-Anne-de-Bellevue (Morgan Arboretum) since 1949 (A. C. Sheppard) and since 1971 (LH), Rougemont since 1971 (LH) and St-Armand since 1982 (LH) and *Herpetogramma aeglealis* has been regularly found at these localities, but *Herpetogramma sphingealis* appeared for the first time in 2003 and in numbers since 2004, suggesting an invading species.

We have not seen any specimens from other provinces in Canada, even from Ontario, although a search of areas where Christmas fern is common may be productive.

#### Remarks.

The genus *Herpetogramma* formerly comprised nine recognized species in North America (Solis 2010), now increased to 10 with *Herpetogramma sphingealis*. The methods used in the present study may prove helpful in future systematic work on the genus.

## Supplementary Material

XML Treatment for Herpetogramma sphingealis

## References

[B1] Dowell P (1911) Notes on ferns attacked by a leaf roller. American Fern Journal 1: 58-59. 10.2307/1544585

[B2] Esaki T Issiki S Mutuura A Inoue I Ogata M Okagaki H Kuroko H (1970) Icones Heterocerum Japonicorum in Coloribus Naturalibus. Hoikusha Publishing Co., Ltd, Osaka, Japan.

[B3] Forbes WTM (1923) The Lepidoptera of New York and Neighboring States, Primitive Forms Microlepidoptera Pyraloids Bombyces. Cornell University Agricultural Experiment Station, Memoir 68. Ithaca, New York.

[B4] Goater B (1986) British Pyralid Moths A Guide to their Identification. Harley Books. Colchester, England.

[B5] Grote AR (1875) IX. North American Pyralides. Bulletin of the Buffalo Society of Natural Sciences 2: 229-232.

[B6] Judd WW (1954) Four species of leaf-tying moths and their dipterous and hymenopterous parasites reared on mayapple, *Podophyllum peltatum* L. Transactions of the American Microscopical Society 73: 401-404. 10.2307/3223585

[B7] Lafontaine JD (2004) Noctuoidea, Noctuidae (part), Noctuinae (part – Agrotini). In: Hodges RW (Ed) The Moths of America North of Mexico fasc. 27.1. The Wedge Entomological Research Foundation, Washington.

[B8] Munroe E (1983) Pyralidae. In: Hodges RW et al. (Eds) Check List of the Lepidoptera of America North of Mexico including Greenland. E. W. Classey Limited and The Wedge Entomological Research Foundation, London.

[B9] Pingyuan W Linyao W Chenglai F Jiuwei B Hongfu Z Youqiao L Xiuqun L Yixin C Guangpu S Baolin Z Zhonglin Z Taoqian H Rongquan C (1981) Iconocraphia [sic] Heterocerum Sinicorum, vol. 1. Science Press, Beijing, China.

[B10] Ruehlmann TE Matthews RW Matthews JR (1988) Roles for structural and temporal shelter-changing by fern-feeding lepidopteran larvae. Oecologia (Berlin) 75: 228-232. 10.1007/BF00378603 28310840

[B11] Slamka F (2010) Pyraloidea of Central Europe / Pyraloidea Mitteleuropas. F. Slamka, Bratislava, Slovakia.

[B12] Solis A (2010) North American *Herpetogramma* Lederer, 1863 (Lepidoptera: Crambidae: Spilomelinae): type specimens and identity of species in the United States and Canada. Proceedings of the Entomological Society of Washington 112: 451-463. 10.4289/0013-8797.112.3.451

[B13] Walker F (1859) List of the Specimens of Lepidopterous Insects in the collection of the British Museum, part XVIII – Pyralides, 510–798.

